# Immobilisation of the Flaps after Amputation

**Published:** 1900-02-10

**Authors:** 


### IMMOBILISATION OF THE FLAPS AFTER AMPUTATION.

Mr. Marmaduke Sheild, of St. George's Hospital, has devised a splint to support the stump after amputation of a limb, more especially after amputation of the thigh. The peculiarity of the splint is that near its extremity it has a large circular opening for the end of the drainage tube. By this arrangement the splint need not be removed at each dressing, as must be done when an ordinary splint is used. The opening for the tube is made through the posterior flap, or, in the case of lateral flaps, the tube is brought out posteriorly. The anterior end of the splint round the aperture is thickly padded with soft iodoform gauze, and, when the flaps have been accurately sutured, the splint is applied next to the limb, and is secured with adhesive strapping and gauze bandages, the end of the tube appearing through the opening in the splint. The usual dressings are then applied, of course enclosing the splint. All the oozing that takes place comes through the tube, and the dressings can easily be changed without disturbing the rest of the limb. We have no doubt that in cases in which the frequent dressings and the flushing of the tube which are referred to are necessary, such a splint as that described will be found very useful, and in any case it will be an improvement of the old plan whenever a drainage tube is used.

